# Structure of the N-Terminal Gyrase B Fragment in Complex with ADP⋅P_i_ Reveals Rigid-Body Motion Induced by ATP Hydrolysis

**DOI:** 10.1371/journal.pone.0107289

**Published:** 2014-09-09

**Authors:** Frédéric V. Stanger, Christoph Dehio, Tilman Schirmer

**Affiliations:** 1 Focal Area Structural Biology and Biophysics, Biozentrum, University of Basel, Basel, Switzerland; 2 Focal Area Infection Biology, Biozentrum, University of Basel, Basel, Switzerland; University of the Basque Country, Spain

## Abstract

Type II DNA topoisomerases are essential enzymes that catalyze topological rearrangement of double-stranded DNA using the free energy generated by ATP hydrolysis. Bacterial DNA gyrase is a prototype of this family and is composed of two subunits (GyrA, GyrB) that form a GyrA_2_GyrB_2_ heterotetramer. The N-terminal 43-kDa fragment of GyrB (GyrB43) from *E. coli* comprising the ATPase and the transducer domains has been studied extensively. The dimeric fragment is competent for ATP hydrolysis and its structure in complex with the substrate analog AMPPNP is known. Here, we have determined the remaining conformational states of the enzyme along the ATP hydrolysis reaction path by solving crystal structures of GyrB43 in complex with ADP⋅BeF_3_, ADP⋅P_i_, and ADP. Upon hydrolysis, the enzyme undergoes an obligatory 12° domain rearrangement to accommodate the 1.5 Å increase in distance between the γ- and β-phosphate of the nucleotide within the sealed binding site at the domain interface. Conserved residues from the QTK loop of the transducer domain (also part of the domain interface) couple the small structural change within the binding site with the rigid body motion. The domain reorientation is reflected in a significant 7 Å increase in the separation of the two transducer domains of the dimer that would embrace one of the DNA segments in full-length gyrase. The observed conformational change is likely to be relevant for the allosteric coordination of ATP hydrolysis with DNA binding, cleavage/re-ligation and/or strand passage.

## Introduction

Type II DNA topoisomerases are essential enzymes that catalyze topological rearrangement of double-stranded DNA (dsDNA) to maintain chromosomes in an appropriate state. In particular, DNA gyrase introduces negative supercoils into covalently closed dsDNA molecules using the free energy generated by ATP hydrolysis. Bacterial DNA gyrase is a prototype of this family composed of two subunits (GyrA, GyrB) and forms a (GyrAGyrB)_2_ dimer of around 400 kDa.

**Figure 1:**
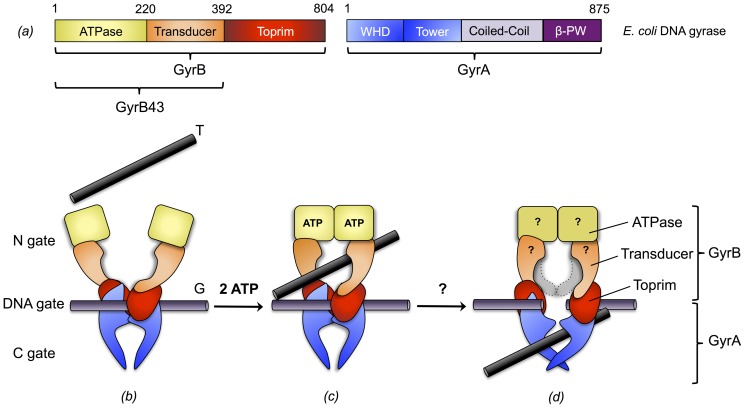
Domain architecture of *E. coli* DNA gyrase and model of DNA gyrase mechanism. (a) Domain architecture of *E. coli* DNA gyrase. GyrB is composed of an ATPase (yellow), a transducer (orange) and a toprim (red) domain. GyrA is composed of a winged-helix (WHD, blue), a tower (blue), a coiled-coil (light purple) and a β-pinwheel (β-PW, purple) domain. (*b*) A double-stranded DNA segment (G-segment) is captured at the DNA-gate, the N-gate is open to allow access of the T-segment. (*c*) Upon ATP binding, the ATPase domains dimerize and the T-segment gets trapped. (*d*) This is followed by ATP hydrolysis, G-segment cleavage, DNA-gate opening and T-segment translocation. The domain colored in light-grey represents the transducer domain in the preceding orientation. The mechanistic details of this step are not clear, in particular whether prior to P_i_ release the enzyme conformation is changed and how ATP hydrolysis and DNA passage are coordinated. For clarity, the coiled-coil and β-pinwheel domains of GyrA are omitted in (b-d). Adopted from references [3], [5].

The dimerization interface is composed of three contact areas or gates (N-, DNA-, C-gate) that open-up successively and in a coordinated fashion to allow DNA passage (Fig. 1). The current view on the enzymatic mechanism is the following two-gate mechanism (reviewed in [1]–[5]). The enzymatic cycle starts by the binding of a segment of double-stranded DNA (gate-segment, G) to the DNA-gate, followed by the trapping of another segment (transfer-segment, T) through the ATP-actuated closure, i.e. dimerization, of the N-gate. Subsequent cleavage of the G-segment and opening of the DNA-gate allows transfer of the T-segment through the gate. Finally, the G-segment gets resealed and the T-segment is released by opening of the C-gate.

The GyrB subunit is comprised of three domains: the N-terminal ATPase domain (GHKL family), the transducer domain, and the C-terminal TOPRIM domain. First insight into the detailed structure of bacterial topoisomerases was obtained with the crystal structure of a 43 kDa N-terminal fragment of *E. coli* GyrB (GyrB43) that comprises the ATPase domain and the central transducer domain [6]. The structure showed a tight dimer with contacts mainly mediated by the ATPase domains (N-gate). The dimer delimits a central hole with a diameter of 20 Å, large enough to accommodate double-stranded DNA (Fig. 1).

The structure was determined in the presence of the substrate analog AMPPNP that is bound to the canonical site of the GHKL-type ATPase domain, but also interacts with the QTK loop of the transducer domain and an N-terminal "brace" of the other subunit of the dimer. Latter interaction explains why only the dimeric form of the GyrB43 fragment is competent for ATP hydrolysis as evidenced by the greater than first-order dependence of the reaction-rate on enzyme concentration [7] and how the presence of ATP stabilizes the closed conformation of the N-gate in GyrB [8].

The different steps of DNA gyrase action have to be tightly coordinated. In particular, G-segment cleavage and translocation of the T-segment should occur only after N-gate closure to prevent non-productive DNA cleavage and to ensure unidirectional segment translocation. Thus, not surprisingly, it could be shown that e.g. ATP hydrolysis is stimulated by the presence of DNA [9]. The question arises whether the ATP hydrolysis event *per se*, i.e. the post-hydrolysis state prior to product release, can be sensed by the enzyme to facilitate or even energize the next catalytic step. In this context it is noteworthy that the substrate is found deeply buried in the GyrB active site without an obvious exit route for the products [6].

Structures of type II topoisomerases have been determined previously for archael topoVI-B' in complex with various nucleotides [10], [11] and for human topoII (htopoII) in complex with AMPPNP and ADP [12], highlighting some of the structural changes occurring along the ATP hydrolysis pathway. Both studies discuss the central role of the conserved lysine (K337 in *E. coli* GyrB) that is proposed to sense the nucleotide state and to relay this information to the center of the enzyme.

Here we report the structures of bacterial GyrB43 in complex with (i) the pre-hydrolysis analog ADP⋅BeF_3_, (ii) the post-hydrolysis ADP⋅P_i_ and (iii) the product ADP. Compared to the AMPPNP complex structure [6], [13], the post-hydrolysis state, i.e. the ternary complex of GyrB43 with ADP and P_i_, shows a substantial domain reorientation, the significance of which is discussed in the context of full-length DNA gyrase.

## Materials and Methods

### Cloning

The gene of *E. coli gyrB* (residues 1-392) was amplified by PCR from *E. coli* genomic DNA using the primer set prFVS107/prFVS114 (5′-GGGAATTCCATATG*CATCACCATCACCATCAC*TCGAATTCTTATGACTCCTCCAG-3′ and 5′-CGACCTCGAGTTAGGTCATTTCACGCGCGCGACG-3′). The DNA sequence corresponding to the N-terminal His_6_-tag is in italics. The amplified DNA fragment was then simultaneously digested with NdeI and XhoI and ligated in the MCS2 of the pRSFDuet-1 vector (Novagen) digested with the same restriction enzymes, resulting in plasmid pFVS0109.

### Protein expression, extraction and purification

The pFVS0109 plasmid was transformed into Ca^2+^-competent *E. coli BL21 (DE3)*. Cells were grown at 37°C in LB medium supplemented with 50 µg/mL of kanamycin and 0.1% glucose to an OD_600_ of 0.6 before induction with 500 µM isopropyl 1-thio-D-galactopyranoside (IPTG) for 5 hours at 37°C. Cells were then harvested by centrifugation. Cells containing overexpressed GyrB43 were resuspended in lysis buffer containing 50 mM Tris pH 7.5, 1 mM EDTA, 5 mM DTT and 5 mM imidazole and disrupted using French press. Cell debris were pelleted by ultracentrifugation at 200 000 *g* and the supernatant was applied to two 1 mL HisTrap column (GE Healthcare) plugged in series. The protein of interest was eluted with a gradient of elution buffer containing 50 mM Tris pH 7.5, 1 mM EDTA, 5 mM DTT and 500 mM imidazole. The eluted fractions containing GyrB protein were then loaded on a 5 mL HiTrap Q column (GE Healthcare) pre-equilibrated with loading buffer (50 mM Tris pH 7.5, 1 mM EDTA, 5 mM DTT). Proteins were eluted with a gradient of elution buffer containing 50 mM Tris pH 7.5, 1 mM EDTA, 5 mM DTT and 1 M NaCl. The protein was then concentrated and injected on a Superdex 75 16/60 gel filtration column (GE Healthcare) equilibrated with 50 mM Na-phosphate pH 7.2, 1 mM EDTA and 5 mM DTT. The different batches of pure protein leading to the crystallization of GyrB in complex with ADP⋅P_i_, ADP⋅BeF_3_ or ADP were concentrated to 36 mg/mL.

### GyrB43/nucleotide complexes crystallization

Crystals were obtained at 20°C using the sitting-drop vapour diffusion method after mixing 0.2 µL protein solution with 0.2 µL reservoir solution equilibrating against a reservoir of 80 µL. For crystallization of GyrB43 in complex with ADP⋅P_i_, the protein solution was prepared by incubating GyrB with a 15 fold molar excess of ATP and MgCl_2_. This allowed the ATPase reaction to start and produce the reaction products: ADP⋅P_i_. GyrB43 in complex with ADP⋅P_i_ crystallized after one week in 0.1 M tri-ammonium citrate pH 6.5 and 25% (w/v) PEG 2000. Crystals were soaked in the reservoir solution supplemented with 20% glycerol for cryoprotection. The protein sample leading to the crystallization of GyrB43 in complex with ADP was prepared similarly to the ADP⋅P_i_ sample. GyrB43 in complex with ADP crystallized after a week in 10% (w/v) PEG 20000, 20% (v/v) PEG MME 550, 0.02 M each carboxylic acid, 0.1 M bicine/Trizma base pH 8.5 (Morpheus screen [14]). For crystallization of GyrB43 in complex with ADP⋅BeF_3_, a prerequisite is the formation of a stable ADP⋅BeF_3_ complex. This was achieved by incubating 20 mM ADP with 500 mM NaF and 100 mM BeCl_2_ for 16 hours at 4°C [8]. The protein solution was prepared by incubating GyrB43 with a 15 fold molar excess of ADP⋅BeF_3_ and MgCl_2_. Crystals grew after a week in 0.2 M NaF, 0.1 M Bis-Tris propane pH 6.5 and 20% (w/v) PEG 3350. Crystals were soaked in the reservoir solution supplemented with 20% glycerol for cryoprotection. All crystals were then flash frozen in liquid nitrogen and stored until data collection.

### Data collection, processing, structure determination and refinement

Diffraction data were collected at the Swiss Light Source (Villigen, Switzerland) at 100 K and processed using XDS [15] and scaled either with XSCALE [15] or aimless [16]. GyrB43 structures were solved by molecular replacement using the previously published structure of the 43-kDa N-terminal fragment of GyrB (PDB entry 1EI1 [13]) as search model using Phaser [17]. Several rounds of iterative model building and refinement were performed using Coot [18] and Refmac5 [19] or PHENIX [20]. 5% of the data were excluded from refinement and used for cross-validation. For the remodeling of the sulfate and asparagine side chain of human topoIIA in complex with ADP⋅SO_4_ (PDB code: 1ZXN), the re-refined model was obtained from the PDB_REDO databank [21]. Iterative model building and refinement was performed as described above. The geometry of the final model was assessed using MolProbity [22] showing >99% of the residues in the core and allowed regions of the Ramachandran plot. Data collections and refinement statistics are summarized in Table 1 and Table 2, respectively.

**Table 1:** Data collection statistics.

	GyrB43 in complex with ADP⋅P_i_	GyrB43 in complex with ADP⋅BeF_3_	GyrB43 in complex with ADP
X-ray source	SLS X06SA (PXI)	SLS X06DA (PXIII)	SLS X06DA (PXIII)
X-ray detector	Pilatus 6M	Pilatus 2M	Pilatus 2M
Wavelength (Å)	1.0000	0.9793	1.0000
Space group	C 2 2 2_1_	C 2 2 2_1_	C 2 2 2_1_
Cell dimensions a, b, c (Å)	77.6, 131.6, 92.4	88.1 143.2 79.9	88.2, 142.5, 79.1
Matthews coefficient (Å^3^Da^−1^)	2.9	3.1	3.1
Solvent content (%)	57.9	59.3	59.3
Molecules in asymmetric unit	1	1	1
Resolution limits (Å)	46.19–1.80 (1.86–1.80)	44.08–2.40 (2.49–2.40)	75.03–2.00 (2.07–2.00)
R_merge_ †(%)	9.9 (112.5)	7.0 (32.8)	7.1 (59.4)
R_meas_ ‡(%)	10.4 (115.5)	7.6 (35.1)	7.7 (67.0)
CC 1/2	99.9 (80.5)	99.9 (97.4)	99.9 (93.7)
<I/σ(I)>	16.4 (2.0)	21.1 (6.1)	21.4 (3.3)
Total reflections	473′415 (42′913)	137′915 (13′976)	192′676 (17′839)
Unique reflections	44′052 (4′261)	20′072 (1′966)	30′053 (3′203)
Wilson B-factor	25.0	36.3	23.5
Multiplicity	10.7 (10.1)	6.9 (7.1)	6.4 (5.6)
Completeness (%)	99.7 (97.4)	99.6 (99.5)	88.2 (95.8)
Mosaicity	0.16	0.46	0.19

Numbers in parentheses belong to the outer shell.

† R_merge_  =  ∑_hkl_∑_i_ |I_i_(hkl) - <I(hkl)>|/∑_hkl_∑_i_ I_i_(hkl), where I_i_(hkl) is the observed intensity for a reflection and <I(hkl)> is the average intensity obtained from multiple observations of symmetry-related reflections.

‡ R_meas_  =  ∑_hkl_ [N/(N-1)]^1/2^ ∑_i_ |I_i_(hkl) - <I(hkl)>|/∑_hkl_∑_i_ I_i_(hkl), where I_i_(hkl) is the observed intensity for a reflection, <I(hkl)> is the average intensity obtained from multiple observations of symmetry-related reflections and N is the number of observations of intensity I(hkl).

**Table 2:** Refinement statistics.

	GyrB43 in complex with ADP⋅P_i_	GyrB43 in complex with ADP⋅BeF_3_	GyrB43 in complex with ADP	htopoII in complex with ADP⋅SO_4_
PDB code	4PRX	4PU9	4PRV	4R1F
Resolution limits (Å)	46.19–1.80 (1.86–1.80)	44.08–2.40 (2.49–2.40)	75.03–2.00 (2.07–2.00)	30.00–2.51 (2.60–2.51)
R_work_ * (%)	16.3 (23.7)	21.9 (28.0)	20.7 (38.5)	20.2 (27.6)
R_free_ **(%)	20.3 (34.2)	27.3 (35.3)	25.9 (43.3)	24.1 (34.5)
Number of non-hydrogen atoms	3′063	2′979	3′015	12′360
* macromolecules*	2′787	2′899	2′861	12′093
* ligands*	39	32	28	133
* solvent*	237	48	126	134
Protein residues	363	374	369	1647
R.m.s.d from ideal				
* Bond lengths (Å)*	0.022	0.016	0.018	0.012
* Bond angles (°)*	2.11	1.97	1.89	1.46
Ramachandran favored *** (%)	98	93	97	95
Ramachandran outliers *** (%)	0.28	0.81	0.27	0.54
Clashscore ***	1.09	4.32	2.13	5.35
Average B values (Å^2^)	30.3	42.2	29.5	64.3
* macromolecules*	30.0	42.4	29.6	64.5
* ligands*	21.3	30.0	22.3	56.0
* solvent*	36.1	40.3	29.8	55.1

Numbers in parentheses refer to the outer shell.

* *R_work_*  =  ∑_hkl_|| F_obs_| - |F_calc_||/∑_hkl_|F_obs_|

** R_free_ is the R value calculated for 5% of the data set that was not included in the refinement.

*** Molprobity.

### Structure analysis

Superimpositions were made using Modtrafo (T. Schirmer unpublished, http://www.biozentrum.unibas.ch/schirmer/modtrafo). Root mean square deviation (RMSD) were calculated using lsqman [23]. To obtain the change in domain orientation, the different structures were superimposed on their respective ATPase domains. Then, the respective transducer domains were superimposed and the resulting rotation matrix analyzed by Modtrafo in terms of polar angles (Ω, φ, κ) with Ω, φ defining the orientation of the rotation axis and κ the rotation angle.

Figures were prepared with Dino (A. Philippsen unpublished, http://www.dino3d.org).

### Accession numbers

Coordinates and structure factors have been deposited in the Protein Data Bank (PDB) with accession numbers 4PRX, 4PU9 and 4PRV for GyrB43⋅ADP⋅P_i_, GyrB43⋅ADP⋅BeF_3_ and GyrB43⋅ADP, respectively. The re-refined and corrected coordinates of htopoII⋅ADP⋅SO_4_ have been deposited in the PDB with accession number 4R1F.

## Results

### Overall structures

To understand better the effect of ATP hydrolysis on the mechanism of GyrB at the atomic level, we aimed to obtain further structures along the reaction pathway, in particular the ADP⋅P_i_ complex structure representing the post-hydrolysis state. A 43 kDa N-terminal fragment of GyrB from *E. coli* (GyrB43) comprising the ATPase and the transducer domain [6], [13] was overexpressed and purified. The fragment was found to be competent for ATP hydrolysis as assessed by an FPLC based nucleotide quantification method [24] yielding k_cat_ and K_m_ values of 2.7⋅10^−3^ s^−1^ and 0.45 mM, respectively. These values are similar to those determined previously for this fragment [7], [13]. GyrB43 was crystallized in presence of ATP substrate (12.5 mM) that was expected to get hydrolyzed to ADP and P_i_. In addition, a high background phosphate concentration (50 mM sodium phosphate buffer) was employed to saturate the γ-phosphate binding site.

**Figure 2:**
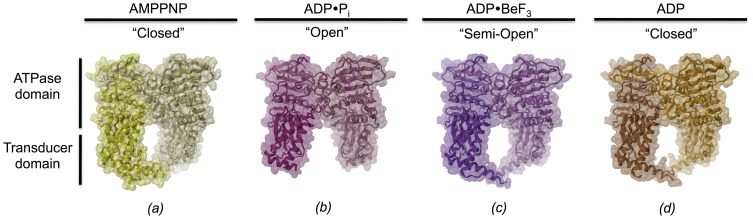
Conformational states of GyrB43 along the ATP hydrolysis reaction path. The various states have been trapped by co-crystallization with the appropriate nucleotides. The dimeric structures are shown in cartoon representation with semi-transparent molecular surface overlaid. Subunits are distinguished by a slight variation in colour hue. View perpendicular to the molecular dyad. (a) AMPPNP complex (PDB entry 1EI1 [13]), (b) ADP⋅P_i_ complex, (c) ADP⋅BeF_3_ complex and (d) ADP complex. Note the distinct opening angles defined by the two transducer domains with the AMPPNP and ADP complexes in a "closed" conformation, the ADP⋅BeF_3_ complex in a "semi-open" and the ADP⋅P_i_ complex in "open" conformation.

Crystals of orthorhombic space group C222_1_ with one monomer per asymmetric unit were obtained that diffracted to 1.8 Å. Data collection statistics are given in Table 1. The structure was solved by molecular replacement using the structure of GyrB43 (*E. coli*) in complex with AMPPNP that had been determined in a different space-group previously (PDB entry 1EI1 [13], Fig. 2a), as search model. The electron density map (2Fo-Fc) calculated with the molecular replacement phases showed well-defined density for the ATPase domain, but rather poor density for the transducer domain. The difference density (Fo-Fc) showed disagreement in the QTK loop region (334–337), which is the part of the transducer domain that extends into the ATP-binding pocket. These observations provided the first indication of a global conformational change.

Rigid- and jelly-body refinement improved the electron density in the transducer domain and caused a drop in the R_free_ from 46.7% to 35.1%. Subsequently, residues 330–340 comprising the QTK loop were rebuilt based on a respective omit-map. An ADP and an adjacent phosphate molecule could be placed unambiguously in the map. Final refinement yielded a GyrB43⋅ADP⋅P_i_ model (Fig. 2b) with R_work_ and R_free_ values of 16.3% and 20.3%, respectively (Table 2). The model comprises all residues from 4 to 378 except segment 304–314 at the tip of the transducer domain.

In addition, the structures of GyrB43 in complex with ADP⋅BeF_3_ (2.4 Å) and in complex with ADP (2.0 Å) were solved. In both cases, plate-like crystals of the same C222_1_ space-group, but with cell constants distinct to those of the GyrB43⋅ADP⋅P_i_ form were obtained. Both structures (Figs. 2c, d) were solved by molecular replacement using the same search model (PDB entry 1EI1) as for the aforementioned structure of the GyrB43⋅ADP⋅P_i_ complex. Again, the ligands could be unambiguously modeled into the Fo-Fc maps. Data collection statistics are given in Table 1. The quality of the electron density maps allowed the tracing of 95% and 94% of the polypeptide chain except segments 305–315, 389–392 for GyrB43⋅ADP⋅BeF_3_ and 303–315, 387–392 for the structure of GyrB43⋅ADP, respectively. Final refinement statistics are given in Table 2.

### Rigid domain reorientations

Structural comparisons of the individual domains of the three newly determined GyrB43 structures in complex with ADP⋅P_i_, ADP⋅BeF_3_ and ADP with the previously published structure of GyrB43⋅AMPPNP reveals that both the transducer and the ATPase domain structures are virtually identical in all complexes with root mean square deviation (rmsd) values between 0.32 and 0.57 Å (Table 3). Still, there are considerable conformational changes between the structures as evident from Fig. 2. In particular, the hole delimited by the two transducer domains of the dimer is larger in GyrB43⋅ADP⋅BeF_3_ and GyrB43⋅ADP⋅P_i_ than in the GyrB43⋅AMPPNP [6], [13] and GyrB43⋅ADP structures.

**Table 3:** Pair-wise fit of GyrB43 domains after superposition of the ATPase domains (regular) or transducer domains (italics).

		rmsd (Å) of ATPase domain (20-220)			
**rmsd (Å) of transducer domain (221**–**392)**		AMPPNP	ADP⋅BeF_3_	ADP⋅P_i_	ADP
AMPPNP			0.422	0.389	0.393
ADP⋅BeF_3_		*0.565*		0.328	0.317
		1.592			
ADP⋅P_i_		*0.391*	*0.423*		0.354
		2.875	1.579		
ADP		*0.501*	*0.369*	*0.354*	
		0.644	1.519	2.891	

The values give the rmsd (Å) of the corresponding Cα positions. When superimposing the ATPase domains dimer, the pairwise rmsd range from 0.46 Å to 0.93 Å.

**Figure 3:**
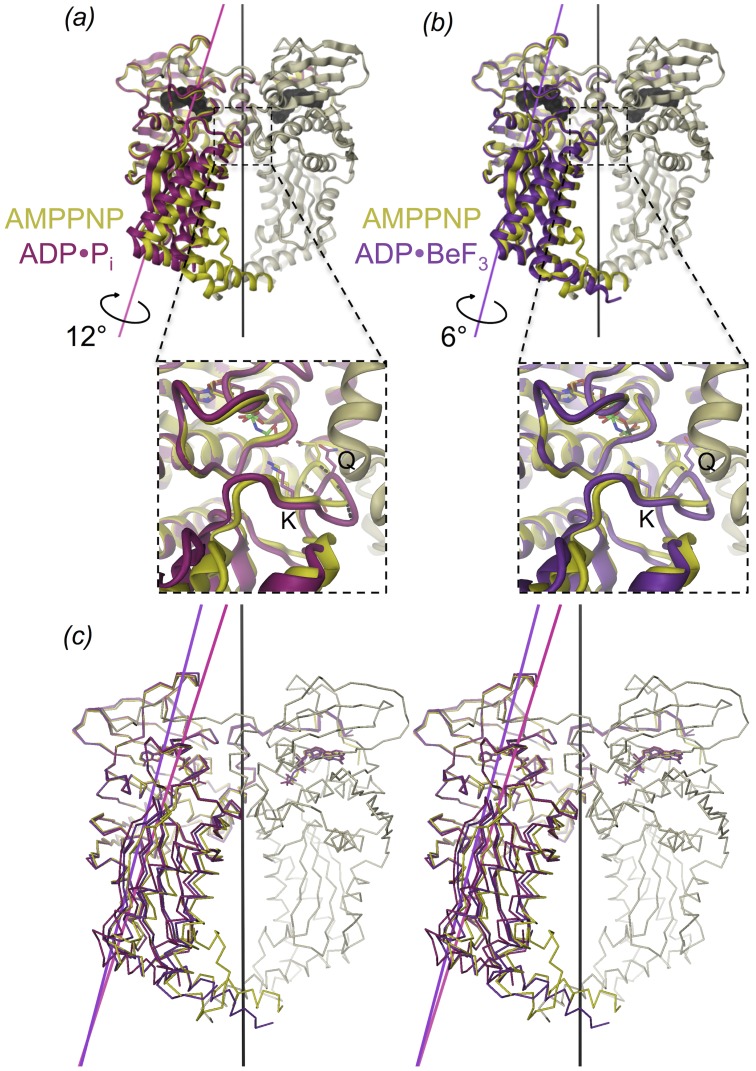
Structure comparison of (a) GyrB43⋅ADP⋅P_i_ (magenta) and (b) GyrB43⋅ADP⋅BeF_3_ (violet) with GyrB43⋅AMPPNP (shown as a dimer with yellow/grey colour with molecular dyad in black). The structures are superimposed on their ATPase domain (residues 20–220). The rotation axes for the domain reorientation of the transducer domain with respect to the ATPase domain are indicated. These form an angle of 21.5° and 14.5° with the molecular dyad for GyrB⋅ADP⋅P_i_ and GyrB⋅ADP⋅BeF_3_, respectively. The insets show the QTK loop of the transducer domain that is rotated relative to the ATPase domain in response to the nucleotide state (dashed lines); Q: Q335, K: K337. (c) Stereoview of the overlay of the three aforementioned structures.

Changes in the arrangement of the two domains are revealed when superimposing the subunits on their ATPase domains (Fig. 3), showing large differences in the respective Cα atoms positions of the transducer domains. The largest rmsd (2.9 Å) occurs between the ternary GyrB43⋅ADP⋅P_i_ complex and the restrained GyrB43⋅AMPPNP substrate complex (Figs. 3a, c and Table 3). The domain re-arrangement is a relative 12° rotation (without translation component) with the rotation axis oriented roughly along the long axis of the monomer and passing through the domain interface (Fig. 3a). Thereby, the transducer β-sheet rolls over the C-terminal helix of the ATPase domain (residues 222 to 232). This part of the interface is mostly hydrophobic. Interacting residues close to the rotation axis are e.g. L197, I222, V226 of the N-terminal and A255, V322 of the C-terminal domain. Interestingly, a rotation about approximately the same axis but by only 6° is needed to superimpose the transducer domain of GyrB43⋅ADP⋅BeF_3_ onto that of GyrB43⋅AMPPNP (Fig. 3b, c). No domain reorientation is observed for the binary GyrB43⋅ADP complex. Table 3 shows that upon superposition of the ATPase domain the transducer domain is still well aligned (rmsd of Cα positions: 0.8 Å).

**Figure 4:**
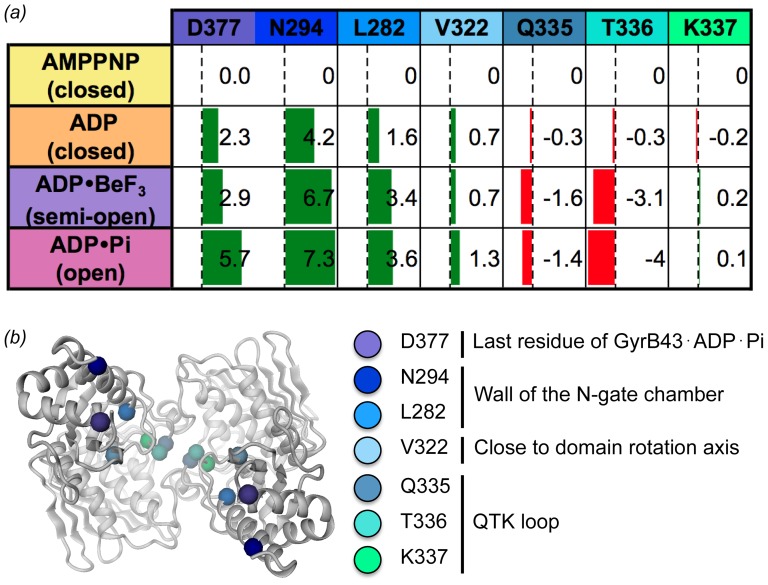
Quantification of nucleotide induced structural changes within dimeric GyrB43. (a) Inter-subunit distance changes between symmetry related residues of selected transducer domain Cα-atoms. The changes have been calculated relative to the AMPPNP complex structure, green bars indicate an increase in distance, red bar indicate a decrease. (b) Cartoon representation of dimeric GyrB43 (ADP⋅P_i_ state) with the residues used for the calculations in (a) indicated. View along the symmetry axis from the C-terminal side.

To further characterize the long-range structural changes, intra-subunit distances were measured within the dimer structures. The changes in the distances are given in Fig. 4. Upon ATP hydrolysis, the QTK loops (Q335 and T336) of the two subunits get closer together at the dimerization interface, whereas residues at the surface of the N-gate chamber (N294, L282) or the C-terminal end (D377) of the transducer domain considerably increase their inter-subunit distance (by up to 7 Å) (see morphing in Movie S1). Accordingly, we define the conformations of the GyrB43⋅ADP⋅BeF_3_ and the GyrB43⋅ADP⋅P_i_ complex "semi-open" and "open", respectively (Fig. 2). Latter structural change may well have relevance for the communication of the ATP hydrolysis event to the core of the gyrase enzyme (see Discussion).

### Crystal packing analysis

In general, variation in protein domain arrangements observed in non-isomorphous crystals can be due to distinct crystal packing forces. Therefore, the various crystal packings were analyzed in detail.

**Figure 5:**
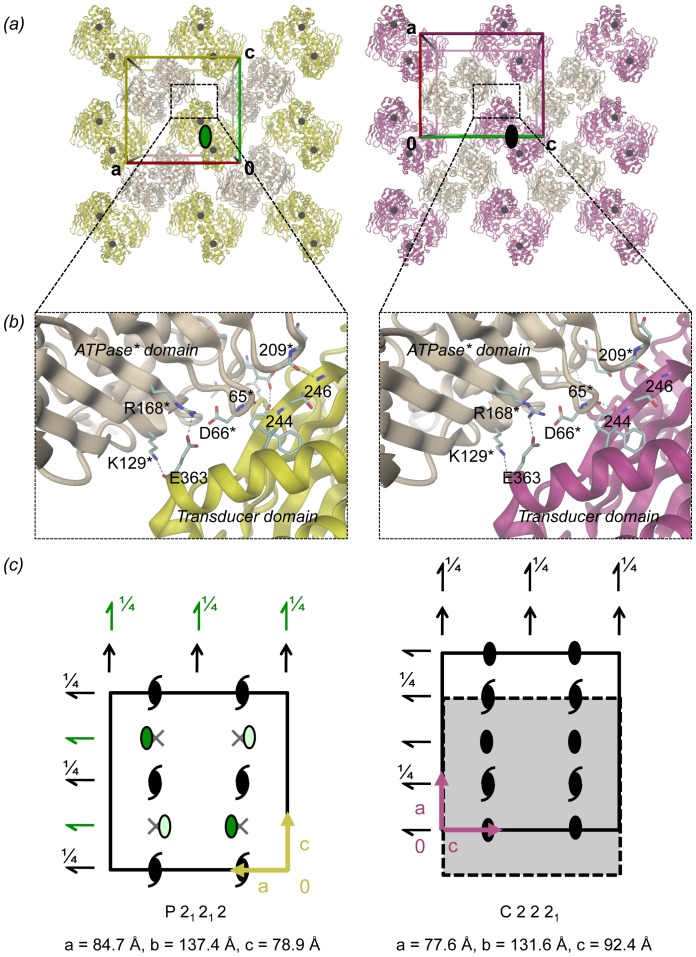
GyrB43 crystal packing of the P2_1_2_1_2 (left, GyrB43⋅AMPPNP complex) and the C222_1_ (right, GyrB43⋅ADP⋅P_i_ complex) form. (a) The molecular packing is shown within a slab perpendicular to b and centered at y = 1/4. In both forms, dimers are oriented with their molecular dyads parallel to b (viewing direction). In the P2_1_2_1_2 form (left) the molecular dyad is local (green elliptical symbol), in the C222_1_ form (right) the molecular dyad is crystallographic (black symbol). In each case, neighbouring dimers are related by horizontal 2-fold screw-axes. Black spheres represent the position of residue D377 at the end of the transducer domain (also depicted in Fig. 4b). (b) Details of the major crystal contact formed in both packings between the transducer domain and the ATPase domain of a symmetry related dimer. (c) Representation of the symmetry elements, same view as in panel (a). Unit cells are indicated by solid line. With respect to the arrangement of symmetry elements, the dashed rectangle of the scheme at the right is equivalent to the unit cell of the left scheme. Local symmetry elements are indicated in green.

The GyrB43⋅ADP⋅P_i_ crystal form is distinct to that of the published GyrB43⋅AMPPNP structure [13], still the molecular packings are related (Fig. 5). Despite the distinct intra-dimer distance between the transducer domains (marked by a black dot in Fig. 5a), GyrB43 dimers are arranged to similar layers (Fig. 5a) due to a conserved ATPase-transducer domain crystal contact with an interface of ∼490 Å^2^ (Fig. 5b). Layers are packed onto each other according to the crystallographic 2_1_ axes along b *via* weak ATPase-ATPase contacts mediated by the "base" of this domain. These interactions are dissimilar between the two crystal forms with a smaller area in the GyrB43⋅AMPPNP form (218 Å^2^) compared to the GyrB43⋅ADP⋅P_i_ form (387 Å^2^). Transducer domains are very weakly or not at all involved in inter-layer contacts in the AMPPNP or ADP⋅P_i_ complex, respectively.

The similarity in packing is reflected in relations between cell parameters/symmetry elements of the two forms (Fig. 5c). In the P2_1_2_1_2 form (GyrB43⋅AMPPNP), the molecular dyad symmetry of GyrB43 is non-crystallographic, whereas, in the C222_1_ form, it coincides with the crystallographic 2-fold symmetry along the long axis (b axis). The positions of local two-fold symmetry axis of GyrB43⋅AMPPNP are depicted in green on Fig. 5c. In space group P2_1_2_1_2, the symmetry is broken by a slight shift of the local symmetry axis along the horizontal direction (Fig. 5c) by 5% to x = 0.29 compared to its theoretical position at a quarter in space group C222_1_.

Concerning the GyrB43⋅ADP complex, the structure was obtained in a crystal form unrelated to that of GyrB43⋅AMPPNP. Still the two structures are virtually identical (see preceding section) ruling out any crystal packing artifacts. Finally, the GyrB43⋅ADP⋅BeF_3_ crystal structure was obtained in a form isomorphous to that of GyrB43⋅ADP. Thus, the observed domain rotation can be attributed to the presence of the BeF_3_ moiety. Altogether, one can conclude that the observed distinct relative orientations of the transducer domains are most likely not due to any crystal packing artifacts, but should be a direct consequence of the distinct complexation states of the ATP pocket.

### Ligand binding

**Figure 6:**
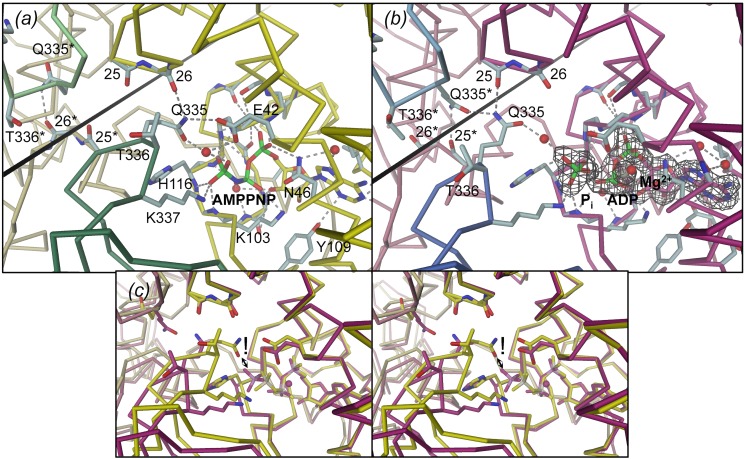
Structures of the GyrB43 nucleotide binding site as determined for (a) the substrate analog complex GyrB43⋅AMPPNP (PDB entry 1EI1 [13]) and (b) the post-hydrolysis complex GyrB43⋅ADP⋅P_i_ with Fo-Fc omit map shown at a contour level of 3.0 σ. Note the distinct interaction of the QTK loop (transducer domain) with the 25–26 loop (ATPase domain) in the two states. The rotation axis for the relative domain reorientation is shown as straight line (same in Fig. 3a) (c) Stereoview of the superimposition of the structures shown in (a) and (b) after superposition on their ATPase domain. The exclamation mark indicates the steric clash that would occur between Q335 in the AMPPNP complex conformation (yellow) with the P_i_ moiety of the post-hydrolysis state (magenta).

**Figure 7:**
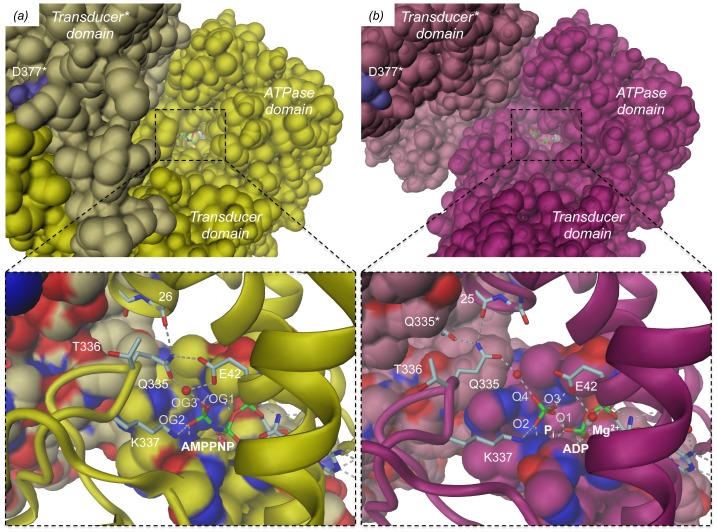
Surface representation of GyrB43 demonstrating the deeply buried nucleotide (stick model). View along the narrow tunnel leading to nucleotide. (a) GyrB43⋅AMPPNP complex, (b) GyrB43⋅ADP⋅P_i_ complex. The insets show close-ups of the nucleotide sites with the ATP lid loop (residues 99–120) and the adjacent subunit of the dimer in surface representation (same colour codes as Fig. 2). Note, that upon ATP hydrolysis glutamine 335 cannot escape "downwards" to relieve a clash with the P_i_ moiety due to the presence of the ATP lid. Instead the entire transducer domain moves to the side.

In the following, we describe in detail the structural changes in GyrB43 that accompany ATP hydrolysis and are coupled to the observed domain orientations. As described first for GyrB43 in complex with AMPPNP [6], [13], the nucleotide is deeply buried in the core of the ATPase domain, with the triphosphate moiety located at the N-terminal end of helix α6 (residues 118–126). The active sites of both *E. coli* GyrB43⋅AMPPNP complex structures [6], [13] are virtually identical (Fig. S1) although the structure determined by Brino *et al*. (PDB code: 1EI1) [13] contains a mutation (Y5S) in the N-terminal arm. Furthermore, inter-species comparison between GyrB from *E. coli* (PDB code: 1EI1) [13] and *Mycobacterium tuberculosis* (PDB code: 3ZKB) [25] reveals a very well conserved ligand-protein interaction network (Fig. S1). Figures 6a and 7a show that the terminal γ-phosphate moiety is held firmly in place with its terminal oxygens forming a multitude of H-bonds with main-chain amide nitrogens of the N-terminus of helix α6 and of the preceding glycine-rich loop. Furthermore, the phosphate forms a salt-bridge with K337 from the transducer domain and interacts indirectly via a water molecule with Q335 (also from the transducer domain) and with E42. Q335, in turn, forms an H-bond with the main-chain carbonyl of residue 26 from the ATPase domain (Fig. 6a and Fig. S3a). In the unreleased GyrB43⋅AMPPNP structure [6], Q335 forms a direct H-bond with the gamma-phosphate of the ligand and lacks the interaction with carbonyl 26.

The structure of GyrB43 in complex with ADP and P_i_ shows the immediate post-hydrolysis state at high resolution (1.8 Å) (Fig. 6b, Fig. S3b and Fig. 7b). Although the distance between the β- and the now liberated distal phosphorus is found significantly increased by 1.5 Å to 4.5 Å, all protein - ligand interactions are virtually unchanged compared to the AMPPNP complex. In particular, despite inverted configuration, two of the oxygens of the phosphate ion (O2, O3) are bonded to the same main-chain amide nitrogens 115–116 and 118–119 as the γ-phosphate in the AMPPNP complex (compare panels (a) and (b) of Figure 6). The phosphate oxygen O3 forms a short H-bond (2.50 Å) with the terminal phosphate of ADP. In the small GTPase Rab11, an analogous interaction (between GDP and P_i_) has been identified by crystallography [26] and by FTIR [27]. The phosphate oxygen O4, the oxygen that evidently has been added upon hydrolysis, projects out of the binding site and interacts with the δ-nitrogen group of H116. Furthermore, it forms a water mediated H-bond with Q335 from the transducer domain. The side-chain amino-group of K337 has moved by 1.0 Å, but is still within H-bonding distance to the now liberated phosphate group. Strikingly, the position of the QTK loop (residues 335–337) with respect to the ligand(s) is considerably distinct in the pre- and post-hydrolysis structure (by about 2.5 to 3 Å) (Fig. 6). This is due to the relative rigid-body motion of the transducer domain described previously. The movement appears to avoid a clash (1.7 Å) that would occur between the Q335 side-chain in AMPPNP complex position and the liberated phosphate group (Fig. 6c). Noteworthy, the side-chain cannot escape sidewise, since its movement is severely restricted by hydrophobic residues of the ATP lid loop (residues 99–120, shown in surface representation in Fig. 7). Rather, the glutamine side-chain amide group finds a new favorable position 3.5 Å apart, where it interacts with its counterpart of the other subunit (Q335*) and main-chain carbonyl 25 of the ATPase domain.

The atom positions of the ADP⋅BeF_3_ ligand in the respective complex are almost indistinguishable to that of the AMPPNP ligand (Fig. S2a compared with Fig. 6a and stereoviews in Fig. S3a compared with Fig. S3c). The substituents of the beryllium atom are arranged tetrahedrally and the beryllium - β-phosphorus distance (2.9 Å) is only marginally shorter than the γ – β-phosphorus distance (3.1 Å). The only major difference is the loss of the (indirect) interaction with Q335 due to the aforementioned transducer domain rotation. Finally, the structure of the GyrB43⋅ADP complex is globally (Table 3) and locally (Fig. S2b and Fig. S3d) almost identical to that of GyrB43⋅AMPPNP (Fig. 6a and Fig. S3a). The only difference is the presence of a water molecule in the approximate position of the absent γ-phosphate. As expected from the unchanged domain organization, the Q335 side-chain interacts with residue 26 in the same way as in the AMPPNP complex structure.

In the three newly solved structures, a magnesium ion is found coordinated by the α- and β-phosphate moieties, one water molecule and the side-chain of N46. In the ADP⋅P_i_ complex, the coordination of the cation seems somewhat distorted to an additional weak interaction with the orthophosphate (O4 - Mg^2+^ distance: 3.1 Å).

Summarizing, the detailed analysis of the ATP binding site revealed pronounced changes in the vicinity of the γ-phosphate subsite upon ATP hydrolysis. How these relate to the observed rigid body motions, which in turn seem crucial for the coordination of gyrase activity will be discussed below.

## Discussion

The endergonic process of negative DNA supercoiling is driven by ATP hydrolysis [28]. ATP binding to the ATPase domain of the GyrB subunit causes N-gate closing and ensures directional transfer of the trapped DNA T-segment through the DNA-gate. Closure of the N-gate may also exert a pinching force that is mediated by the transducer domains on the trapped DNA segment to facilitate transfer through the DNA-gate [3]. It has been demonstrated that ATP hydrolysis occurs sequentially for yeast topoisomerase II [29]. Hydrolysis of the first ATP molecule is sufficient for the catalysis of DNA cross-passage, while only upon hydrolysis of the second ATP molecule the ATPase domains dissociate to reset the enzyme. In contrast, *B. subtilis* GyrB appears to hydrolyze ATP synchronously [30]. Considering the close homology, a similar mechanism would be expected also for bacterial DNA gyrase.

Here, we have studied the nucleotide-state dependent conformations of GyrB43 prior to nucleotide release, i.e. prior to ATPase domain dissociation, with the aim to provide further and detailed structural information about the coordination of ATP hydrolysis and strand passage.

### Nucleotide-dependent topoisomerase II conformations

The triphosphate-nucleotide complexed conformations of the ATPase/topoisomerase fragments match closely for the bacterial [6], [13], eukaryotic [12], and archaeal [10], [11] structures. It has been dubbed "restrained" conformation [11] due to the tight packing of the transducer domain against the ATPase domain and the multiple interactions of the ligand with the ATP binding site. These include interactions with the ATP lid loop, the N-terminal segment of adjacent subunit and the glycine rich loop that tightly embraces the γ-phosphate. Moreover, residues of the QTK loop (being part the transducer domain) are also engaged in ATP binding. For the slightly truncated subunit B of archaeal topoisomerase VI (topoVI-B'), comparison of the restrained (dimeric) AMPPNP complex with the "relaxed" (monomeric) nucleotide-free state revealed a relative reorientation of the two domains [11] giving a first hint for the potential of allosteric communication via these domains. In the dimeric structures of topoVI- B', the ADP, ADP⋅P_i_ and the ADP⋅AlF_4_ complexes all showed the restrained conformation [11]. This suggested that ATP hydrolysis would elicit no large-scale structural response in topoVI.

In contrast, human topoisomerase II shows a semi-open ADP complex characterized by an 8° outward rotation of the transducer domain when compared with its restrained/closed AMPPNP conformation [12] (Fig. S4a). Closer inspection and re-refinement of the original structure (PDB code: 1ZXN), however, revealed that the γ-phosphate binding subsite is not empty, but occupied by a sulfate ion in 3 of the 4 molecules of the asymmetric unit (the active site of the fourth molecule contains a glycerol molecule). The corrected structure (Fig. S4b) with improved statistics (R_work_/R_free_ (%) of 20.2/24.1) has been deposited in the Protein Data Bank (PDB code: 4R1F). Thus, the structure apparently represents an ADP⋅SO_4_ mimic of the post-hydrolysis state and the domain rotation may well be of functional relevance (Fig. S4, see also below).

For bacterial gyrase, all of the conformational states along the ATP hydrolysis pathway are now known. The canonical, restrained structure is attained both in presence of AMPPNP [6], [13] and ADP, as for topoVI-B'. This indicates that the γ-phosphate is not required to restrain the conformation of the enzyme. The effect of ATP hydrolysis would then be the enhancement of nucleotide dissociation to reset the enzyme to the monomeric ATPase state [5]. This is in contrast to, e.g., small GTPases that act like a "loaded spring" and switch to a relaxed state upon GTP hydrolysis and phosphate release [31].

Here, we have shown for the bacterial GyrB43 fragment that there is an additional effect of ATP hydrolysis that occurs prior to phosphate release and may be of functional significance. The ADP⋅P_i_ post-hydrolysis state is characterized by a virtually unchanged ATPase dimer structure, but with the transducer domains reoriented as rigid bodies by 12° relative to their position in the substrate analog complex (see morphing in Movie S1).

Interestingly, also human topoII may undergo a similar structural transition as inferred by the comparison of the respective AMPPNP and ADP⋅SO_4_ complexes (Fig. S4). As for bacterial gyrase, the pivot of the motion is at the center of the interface between the C-terminal ATPase domain helix and the transducer β-sheet, but the direction of the rotation axis is different (Fig. S4a). In contrast, the archaeal topoisomerase IIB enzyme topoVI exhibits no structural change upon ATP hydrolysis [11]. This can be attributed to a distinct QTK loop with only the lysine conserved in this distantly related topoisomerase family (Fig. S5), see below.

The conformational change observed in GyrB43 can be traced back to the significant, but small (1.5 Å) increase in the distance between the β-phosphate and the terminal γ-phosphate or phosphate moiety, respectively. The direct structural consequences of the shifted γ-phosphate position on the local organization of the binding site and particularly on the position of the QTK loop will be discussed in the following section.

### The central role of the QTK loop in bacterial and eukaryotic topoisomerases (topoisomerases IIA)

All topoisomerases IIA studied so far adopt the restrained/closed conformation in complex with tri-phosphate substrate analogs. In contrast, the post-hydrolysis complex has adopted the open state, since the phosphate would severely clash with Q335 from the QTK loop of the transducer domain in the restrained state (Fig. 6c). Close inspection of the restrained structure reveals that such strain cannot be relieved by a simple side-chain rotation of the tightly buried glutamine. Also, there is no apparent route for the phosphate to leave the binding site, since the previously described tunnel [13] seems too narrow (Fig. 7). Instead, the observed movement of the entire transducer domain appears mandatory to accommodate the post-hydrolysis state in which the side-chain of Q335 forms a new set of H-bonds, i.e. with its symmetry mate Q335* and a main-chain carbonyl of the ATPase domain.

Thus, Q335 appears to fulfill a central role by acting as a two-state switch in response to the nucleotide state of the ATPase domain. Indeed, an early biochemical *in vivo* study on *E. coli* gyrase [9] had already identified this glutamine as indispensable for the regulation of ATP hydrolysis by DNA binding. A later study on a QTK deletion mutant of human topoII again showed deregulation of the enzyme with DNA cleavage no longer controlled by the nucleotide state of the enzyme [32].

The equivalent of the QTK loop in archaea carries no glutamine (Fig. S5) and exhibits a distinct main-chain geometry [10]. Therefore, the nucleotide-binding pocket is considerably more spacious. This explains why the post-hydrolysis state can be accommodated in the restrained conformation and why no ATP hydrolysis induced structural changes have been observed [11]. Whether also in archaeal topoisomerases the event of ATP hydrolysis can be signaled to the core of the enzyme remains to be investigated.

### Coordination of ATP hydrolysis and supercoiling activity

Modification of DNA topology needs the coordinated catalysis of various steps. Therefore, it is not surprising that DNA binding positively affects ATPase activity as shown for *E. coli* gyrase [33]. The simplest explanation for this allosteric regulation is that T-segment binding to the N-gate chamber of gyrase induces a change in the relative disposition of the two transducer domains which in turn is coupled to local changes in the ATPase active site [29]. Since the transducer domain carries the active lysine (K337), a reorientation of the transducer domain with respect to the ATPase dimer with the bound ATP substrates should indeed affect ATP hydrolysis.

Such DNA induced domain reorientation has, to our knowledge, not yet been observed directly. It is likely, however, that for such communication the in-built domain mobility described in detail here is exploited again. In the restrained state, the side-chain amino-group of K337 is in H-bonding distance to the γ-phosphate of the substrate analog and, thus, probably capable to stabilize the negative charge of the transition state [9]. In the other, open conformation of the enzyme, the K337 amino-group is pulled out of the nucleotide pocket by 1.0 Å, probably sufficient to significantly de-tune the enzyme [9].

**Figure 8:**
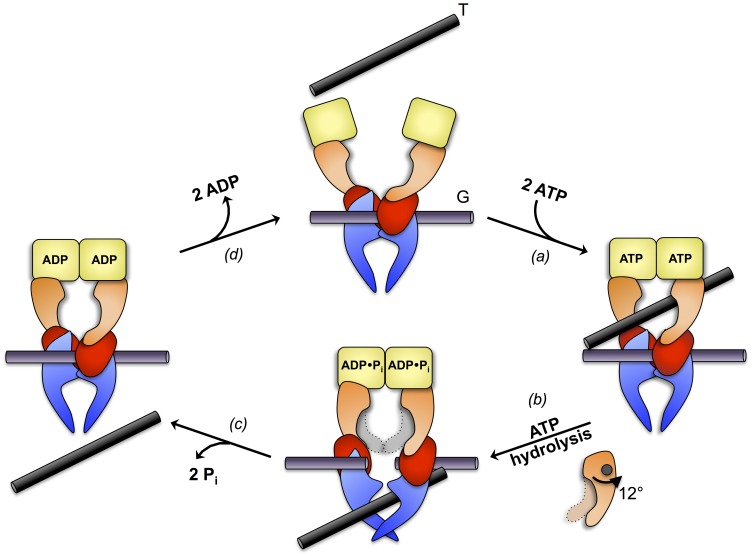
Refined mechanistic scheme of DNA gyrase activity, based on reference [2], representation as in **Fig. 1.** The N-gate of the GyrA_2_GyrB_2_ heterotetramer with bound G-segment at the central DNA-gate (top) closes upon ATP binding thereby trapping a T-segment in the upper chamber (step a). Hydrolysis of the two ATP molecules causes a 12° rotation of the respective transducer domains relative to the ATPase domain (step b). We propose that this conformational change is coupled to DNA gate opening and T-segment translocation. Subsequent P_i_ release would be coordinated with G-segment re-ligation and DNA-gate closure (step c). Finally, ADP release results in dissociation of the ATPase domains and a reset of the enzyme (step d).

Is there signaling also in the opposite direction, from the active sites of the ATPase dimer to the core of the enzyme? This could then synchronize ATP hydrolysis with the other catalytic steps. Here, we have shown by detailed structural studies that prior to phosphate release a well-defined, obligatory state is attained and that this post-hydrolysis state is characterized by a significantly increased distance between the distal transducer ends of the GyrB43 fragment. We propose that this ATP hydrolysis induced movement initiates a series of events starting with G-segment cleavage that would be followed by DNA-gate opening and T-segment passage. The refined mechanistic scheme for the enzymatic cycle of full-length gyrase based on the current two-gate model [2] is shown in Fig. 8. It has been suggested that part of the free energy generated by ATP hydrolysis may be used to actively push the T-segment through the DNA gate [3]. This would indeed be consistent with the observed rigid-body rearrangement.

## Supporting Information

Figure S1 **Comparison of various GyrB structures in complex with AMPPNP.** (a) Superimposition of the *E. coli* GyrB43⋅AMPPNP structure determined by Wigley *et al*. (unreleased, personal communication, note that AMPPNP had been modeled as ATP) [6] in brown onto our reference structure *E. coli* GyrB43⋅AMPPNP determined by Brino *et al*. (PDB code: 1EI1) [13] in yellow. (b) Superimposition of *M. tuberculosis* GyrB⋅AMPPNP in blue-green onto our reference structure *E. coli* GyrB43⋅AMPPNP (PDB code: 1EI1) [13] in yellow. Structure of the ligand binding site of (c) *E. coli* GyrB⋅AMPPNP determined by Wigley *et al*. and (d) *M. tuberculosis* GyrB⋅AMPPNP (PDB code: 3ZKB). Hydrogen bonds are depicted as grey dashed lines. (e) Close-up stereoview of the active sites of the two structures shown in (a). (f) Close-up stereoview of the active sites of the two structures shown in (b).
(TIF)

Figure S2 **Details of the binding sites of GyrB43 in complex (a) with ADP⋅BeF_3_ and (b) ADP.** H-bonds are depicted by dashed grey lines. The Fo-Fc omit electron density maps are shown at a contouring level of 3.0 sigma.
(TIF)

Figure S3 **Stereoviews of the details of the binding sites of GyrB43 in complex with (a) AMPPNP, (b) ADP⋅P_i_, (c) ADP⋅BeF_3_ and (d) ADP.** H-bonds are depicted by dashed grey lines. The Fo-Fc omit electron density maps are shown at a contouring level of 3.0 sigma.
(TIF)

Figure S4 (a) Superimposition of the human topoII in complex with AMPPNP (PDB entry 1ZXM, dark blue) and in complex with ADP (light blue) [12]. (b) Active site details of htopoII in complex with AMPPNP (left) or ADP⋅SO_4_ (right), with the Fo-Fc omit electron density map for ADP, SO_4_ and Mg^2+^ shown at a contouring level of 3.0 sigma. (c) Stereoview of the structures shown in (b) after superposition on their ATPase domain. The exclamation mark indicates the steric clash that would occur between Q376 in the AMPPNP complex conformation (dark blue) with the SO_4_ moiety of the post-hydrolysis mimic state (light blue).
(TIF)

Figure S5 **Sequence alignment of the region encompassing the QTK loop from representative species of bacteria, eukaryotes and archaea.** The QTK loop is strictly conserved in topoIIA but absent from topoIIB.
(TIF)

Movie S1 **Morphing between the GyrB43⋅AMPPNP complex (PDB entry 1EI1** [13] **) and the post-hydrolysis complex GyrB43⋅ADP⋅P_i_, shown in cartoon representation with semi-transparent molecular surface overlaid with the same colors as in** **Fig. 2** **(AMPPNP state in yellow and ADP⋅P_i_ state in magenta).** The molecular two-fold axis is shown as a black line. Views perpendicular (left) and along (right) the molecular dyad.
(MOV)
