# Supplementary figures and images for: Structure of the N-Terminal Gyrase B Fragment in Complex with ADP⋅Pi Reveals Rigid-Body Motion Induced by ATP Hydrolysis
Source: PLoS One. 2014 Sep 9;9(9):e107289. doi: 10.1371/journal.pone.0107289 (PMC4159350; doi:10.1371/journal.pone.0107289)

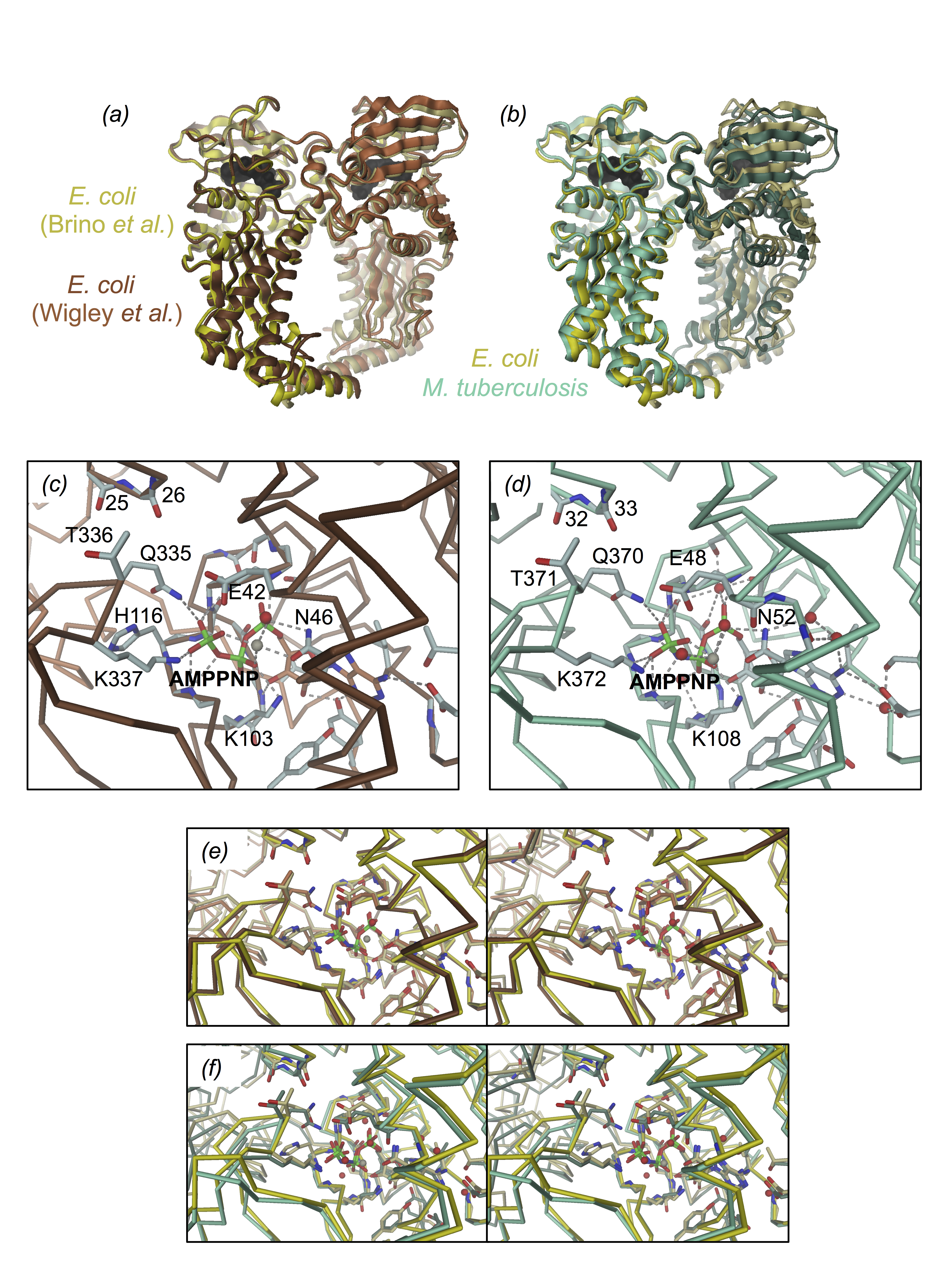

Supplement: Figure S1 — Comparison of various GyrB structures in complex with AMPPNP. (a) Superimposition of the E. coli GyrB43⋅AMPPNP structure determined by Wigley et al. (unreleased, personal communication, note that AMPPNP had been modeled as ATP) [6] in brown onto our reference structure E. coli GyrB43⋅AMPPNP determined by Brino et al. (PDB code: 1EI1) [13] in yellow. (b) Superimposition of M. tuberculosis GyrB⋅AMPPNP in blue-green onto our reference structure E. coli GyrB43⋅AMPPNP (PDB code: 1EI1) [13] in yellow. Structure of the ligand binding site of (c) E. coli GyrB⋅AMPPNP determined by Wigley et al. and (d) M. tuberculosis GyrB⋅AMPPNP (PDB code: 3ZKB). Hydrogen bonds are depicted as grey dashed lines. (e) Close-up stereoview of the active sites of the two structures shown in (a). (f) Close-up stereoview of the active sites of the two structures shown in (b). (TIF) [file pone.0107289.s001.tif]

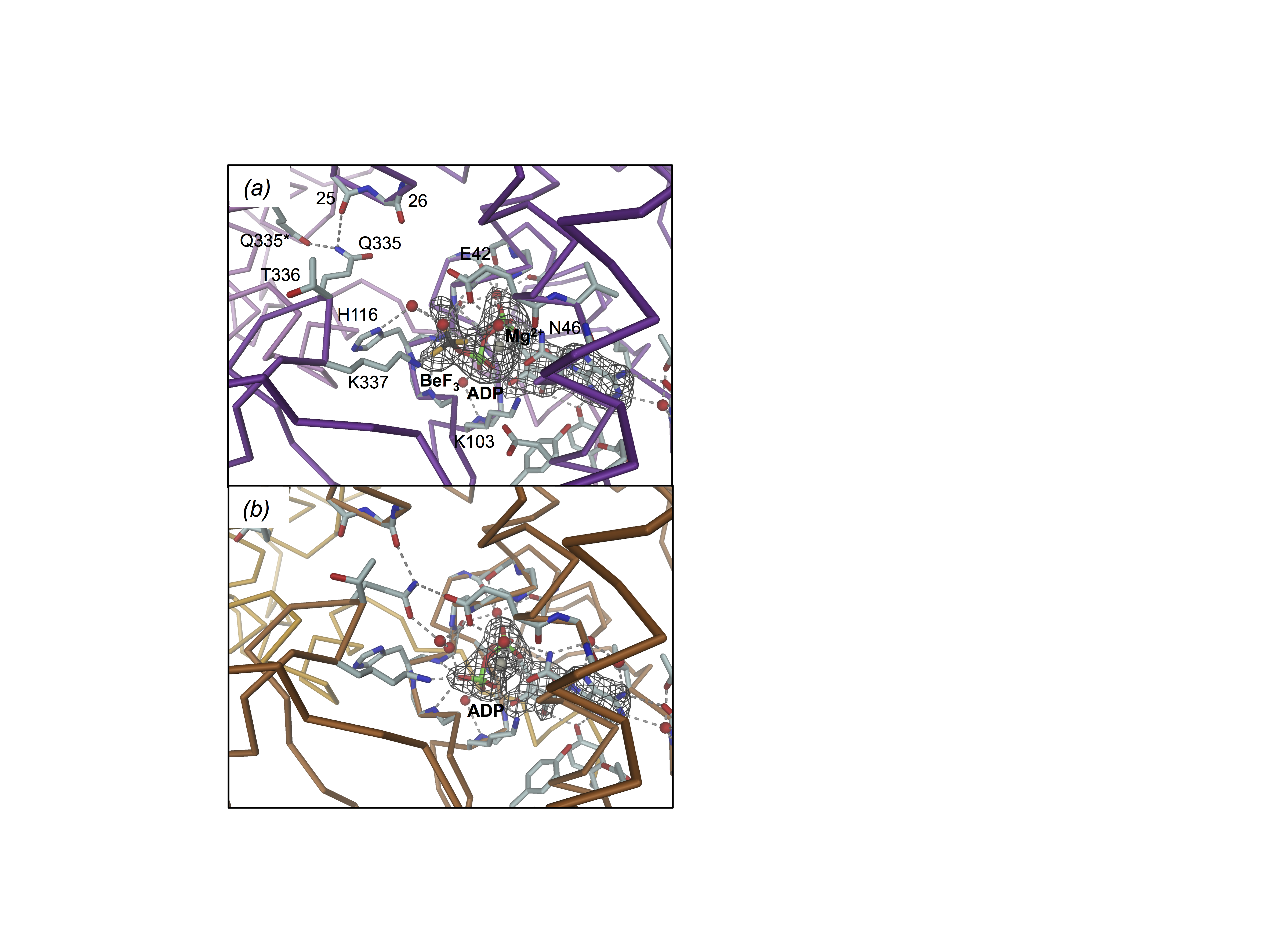

Supplement: Figure S2 — Details of the binding sites of GyrB43 in complex (a) with ADP⋅BeF3 and (b) ADP. H-bonds are depicted by dashed grey lines. The Fo-Fc omit electron density maps are shown at a contouring level of 3.0 sigma. (TIF) [file pone.0107289.s002.tif]

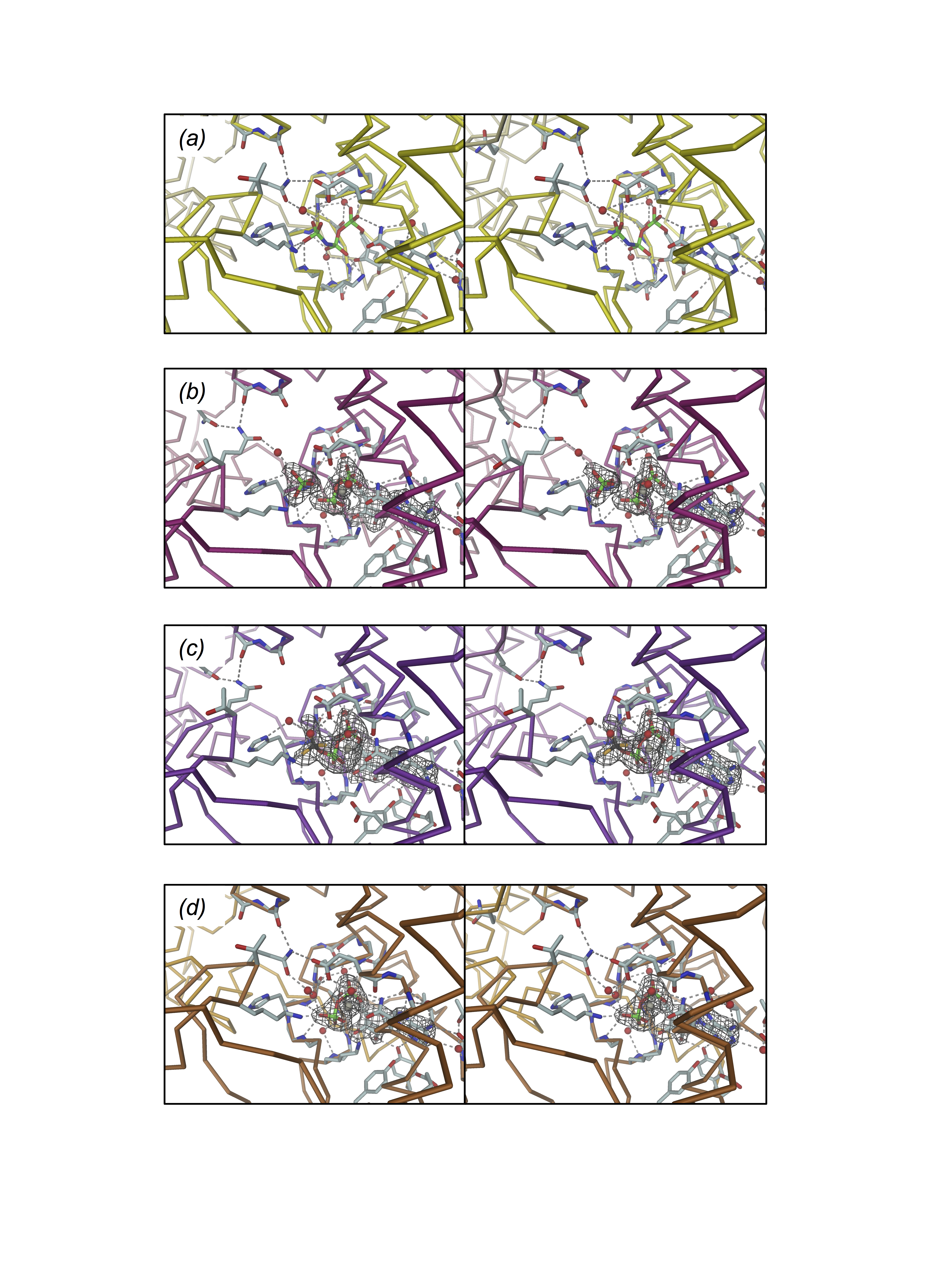

Supplement: Figure S3 — Stereoviews of the details of the binding sites of GyrB43 in complex with (a) AMPPNP, (b) ADP⋅Pi, (c) ADP⋅BeF3 and (d) ADP. H-bonds are depicted by dashed grey lines. The Fo-Fc omit electron density maps are shown at a contouring level of 3.0 sigma. (TIF) [file pone.0107289.s003.tif]

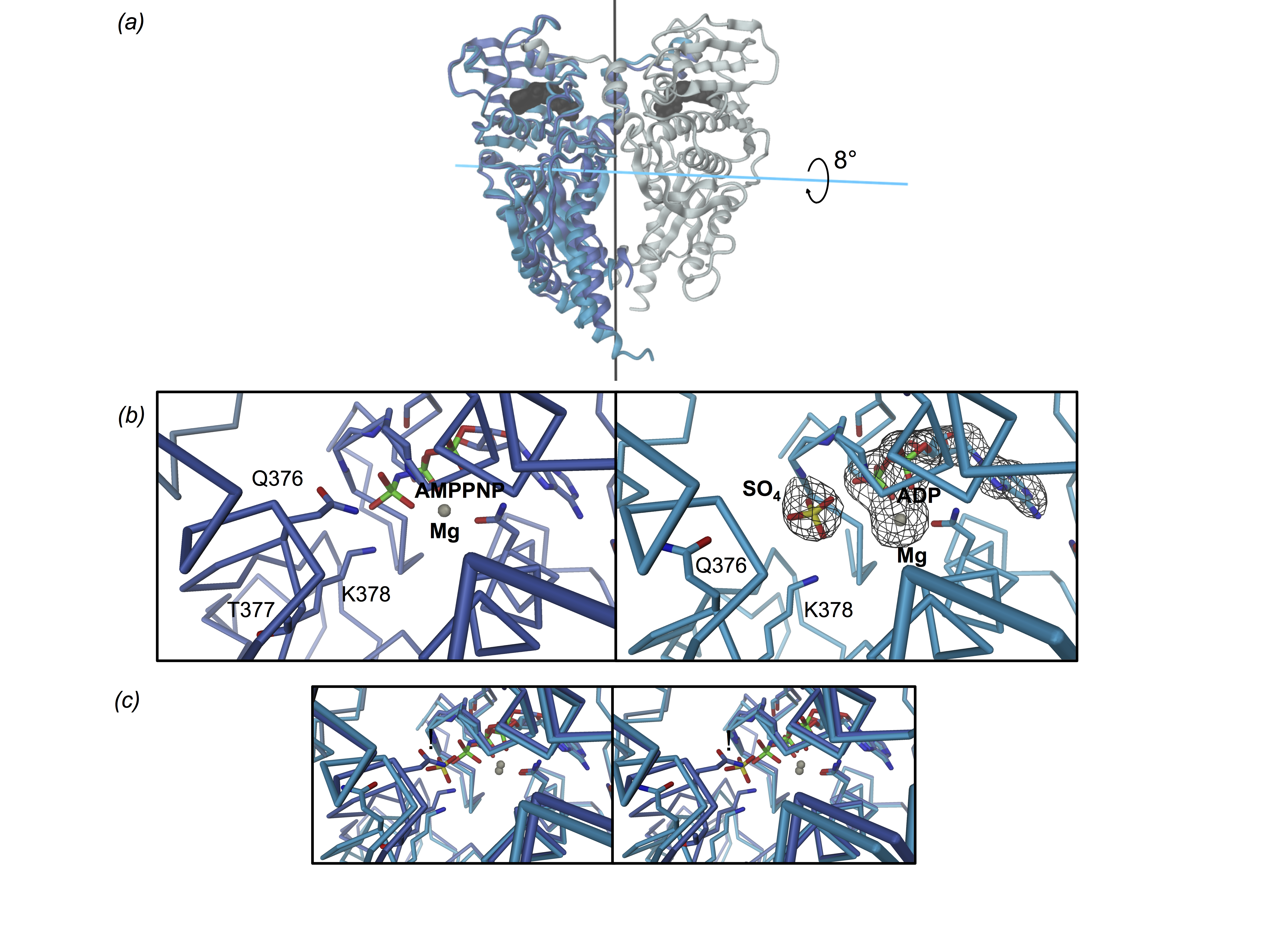

Supplement: Figure S4 — (a) Superimposition of the human topoII in complex with AMPPNP (PDB entry 1ZXM, dark blue) and in complex with ADP (light blue) [12]. (b) Active site details of htopoII in complex with AMPPNP (left) or ADP⋅SO4 (right), with the Fo-Fc omit electron density map for ADP, SO4 and Mg2+ shown at a contouring level of 3.0 sigma. (c) Stereoview of the structures shown in (b) after superposition on their ATPase domain. The exclamation mark indicates the steric clash that would occur between Q376 in the AMPPNP complex conformation (dark blue) with the SO4 moiety of the post-hydrolysis mimic state (light blue). (TIF) [file pone.0107289.s004.tif]

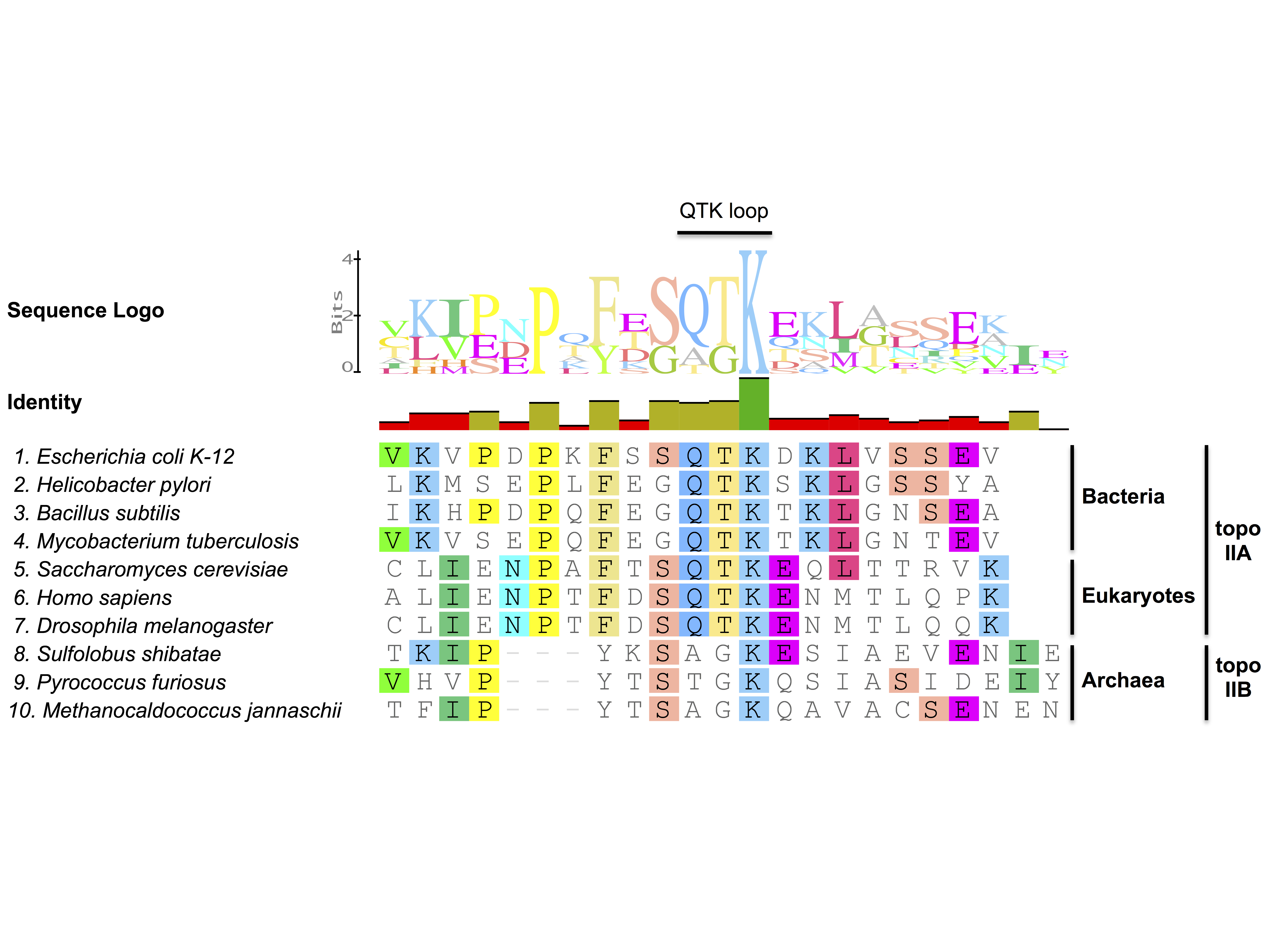

Supplement: Figure S5 — Sequence alignment of the region encompassing the QTK loop from representative species of bacteria, eukaryotes and archaea. The QTK loop is strictly conserved in topoIIA but absent from topoIIB. (TIF) [file pone.0107289.s005.tif]
